# Oral Sensory Neurons of the Geniculate Ganglion That Express Tyrosine Hydroxylase Comprise a Subpopulation That Contacts Type II and Type III Taste Bud Cells

**DOI:** 10.1523/ENEURO.0523-21.2022

**Published:** 2022-10-12

**Authors:** Tao Tang, Brian A. Pierchala

**Affiliations:** Department of Anatomy, Cell Biology & Physiology, Indiana University School of Medicine, Stark Neurosciences Research Institute, Indianapolis, IN 46202

**Keywords:** taste, tyrosine hydroxylase, geniculate ganglion, chemosensory neuron, taste bud, gustatory

## Abstract

Oral sensory neurons of the geniculate ganglion (GG) innervate taste papillae and buds on the tongue and soft palate. Electrophysiological recordings of these neurons and fibers revealed complexity in the number of unique response profiles observed, suggesting there are several distinct neuronal subtypes. Molecular descriptions of these subpopulations are incomplete. We report here the identification of a subpopulation of GG oral sensory neurons in mice by expression of tyrosine hydroxylase (TH). TH-expressing geniculate neurons represent 10–20% of oral sensory neurons and these neurons innervate taste buds in fungiform and anterior foliate taste papillae on the surface of the tongue, as well as taste buds in the soft palate. While 35–50% of taste buds on the tongue are innervated by these TH+ neurons, 100% of soft palate taste buds are innervated. These neurons did not have extragemmal processes outside of taste buds and did not express the mechanosensory neuron-associated gene *Ret*, suggesting they are chemosensory and not somatosensory neurons. Within taste buds, TH-expressing fibers contacted both Type II and Type III cells, raising the possibility that they are responsive to more than one taste quality. During this analysis we also identified a rare TH+ taste receptor cell type that was found in only 12–25% of taste buds and co-expressed TRPM5, suggesting it was a Type II cell. Taken together, TH-expressing GG oral sensory neurons innervate taste buds preferentially in the soft palate and contact Type II and Type III taste bud receptor cells.

## Significance Statement

Impaired taste sensation is a consequence of neurodegenerative diseases, chemotherapy, viral infections and normal aging. Understanding how oral sensory information is conveyed from the mouth to the brain is necessary for development of therapies for taste impairments. Our understanding of the molecular underpinnings of peripheral oral sensory neuron populations that convey chemosensory, thermal and tactile information from the oral cavity to the brainstem is rudimentary. We identified a small subpopulation of geniculate and petrosal ganglion oral sensory neurons that express tyrosine hydroxylase (TH) and project into taste buds in all regions of the oral cavity. TH-expressing neurons contacted two different classes or taste bud cells, Type II and Type III cells, suggesting they may respond to more than one taste quality.

## Introduction

Feeding is a life-essential behavior that requires the coordinated activities of chemosensory neurons responsible for taste and olfaction, along with somatosensory neurons. These varied modalities are communicated by neurons from peripheral ganglia whose afferent projections converge at higher order CNS centers to create the complex sensations of flavor (Small, 2012; Samuelsen and Vincis, 2021). Feeding also requires critical motor components for chewing, swallowing and the protection of the larynx and airways (Hiiemae and Palmer, 2003; Steele and Miller, 2010; Westberg and Kolta, 2011; Prescott et al., 2020). The peripheral taste system is composed of sensory organs, the taste buds, that in mice are located in several types of papillae located on the tongue, along with taste buds located in the soft palate and larynx that are not in papillae. The cell bodies of sensory neurons that innervate them are located in the geniculate ganglion (GG) and nodose/petrosal/jugular ganglion complex. Our understanding of how specific taste qualities detected by taste buds are communicated to the CNS, and how these signals are interpreted to affect eating behaviors, is rudimentary.

What is known about the oral sensory neurons of the geniculate and nodose/petrosal/jugular ganglia is limited, with most studies examining the GG because of its somewhat simpler anatomy. Oral sensory neurons of the GG project either to fungiform or foliate taste buds in the tongue via the chorda tympani nerve, or to taste buds in the soft palate via the greater superficial petrosal nerve. Importantly, recent studies have highlighted the fact that geniculate neurons that innervate fungiform papillae respond to both chemical stimuli as well as tactile and temperature (cold) stimuli (Biedenbach and Chan, 1971; Finger et al., 2005; Breza et al., 2006; Yokota and Bradley, 2016, 2017; Kumari et al., 2017; Donnelly et al., 2018), making fungiform papillae sensory organs that may integrate several modalities (Mistretta and Bradley, 2021). Measurements of activity of individual oral sensory neurons and fibers indicate that there is an assortment of unique subtypes of neurons, with some neurons only responding to one tastant (specialists) and others being more broadly tuned (generalists; Frank et al., 2008; Ohla et al., 2019). The accompanying information regarding their molecular (Dvoryanchikov et al., 2017; J Zhang et al., 2019) and morphologic diversity (T Huang et al., 2021) is just beginning to emerge.

One method to identify specific subclasses of neurons within a given population is to examine their neurotransmitter and neuropeptide phenotype. One neurotransmitter class that has been useful in this regard is the catecholamines, which has been evaluated by the expression of their synthesis enzymes. Distinct subtypes of catecholaminergic neurons underlie several important behaviors such as motor control pathways, circuits involved in motivation and reward, and peripheral sympathetic neurons involved in homeostasis (Ikemoto, 2007; Ugrumov, 2009; Zhu et al., 2012). The most frequently used “marker” of this pathway is tyrosine hydroxylase (TH), which is the first and main rate-limiting enzyme for the synthesis of catecholamines. TH-expressing fibers have been reported to innervate circumvallate taste buds (intragemmal fibers; Dvoryanchikov et al., 2007), but in other regions of the oral cavity TH+ fibers are absent from taste buds (Yamamoto et al., 1997; Ohman-Gault et al., 2017). TH-expressing fibers have also been reported to project near taste buds within taste papillae (extragemmal fibers) but not enter the taste bud itself (Ohman-Gault et al., 2017). Examination of TBs for the presence of catecholamine production revealed that TBs appear capable of making norepinephrine, although TH itself was not observed in TB cells (Dvoryanchikov et al., 2007; YA Huang et al., 2008).

We used sensitive and specific molecular genetic tools to determine whether there is a population of TH+ oral sensory neurons in the GG, and, if identified, evaluate the morphology of their terminal endings in taste papillae. We identified a small group of TH+ neurons in the GG that innervated fungiform, foliate and soft palate taste buds, and did not have extragemmal projections. Within taste buds, these neurons contacted both Type II and Type III taste receptor cells. In addition, we observed a rare TH+ taste receptor cell in a small percentage of fungiform and circumvallate taste buds that co-labeled with TRPM5, a marker of Type II cells.

## Materials and Methods

### Animals

Adult, postnatal day (P)90 wild-type C57BL6/J mice were used in real-time PCR and *in situ* hybridization experiments to determine the expression level of *Th* in the geniculate and trigeminal ganglia (TG). Rosa26^LSL-TdTomato/+^ mice (The Jackson Laboratory, Ai14, stock #007914) were crossed with mice harboring the CreERT2-mediated recombination system driven by the *Th* promoter (*Th*-CreER, Harvard University) to generate *Th*^CreER/+^; Rosa26^LSL-TdTomato/+^ mice and *Th*^+/+^: Rosa26^LSL-TdTomato/+^ control mice. These mice were used as a reporter line to monitor expression of *Th* via RFP. Dual-expression reporter mice for *Phox2b* and *Th* were produced by crossing mice which have the RC::FLTG dual-recombinase responsive indicator allele (The Jackson Laboratory, stock #026932) with mice harboring the *Th^CreER^* transgene to generate *Th^CreER/+^*; RC::FLTG mice. These were then crossed with mice carrying the FLP recombinase system under control of the Phox2b promoter (*Phox2b^FLPo^*, The Jackson Laboratory, stock #022407) to generate *Th^CreER/+^*; *Phox2b^Flpo/+^*; RC::FLTG mice. In these triple transgenic mice GFP is only present in neurons that express both *Th* and *Phox2b*. All experiments were performed in compliance with the guidelines of the American Association for Accreditation of Laboratory Animal Care International (AAALAC) and were approved by the Institutional Animal Care and Use Committees of the Institutions. Mice were group housed whenever possible, were provided environmental enrichment, and were on a 12/12 h light/dark cycle. Equal numbers of male and female mice were examined in all experiments.

### Tamoxifen administration

Mice received tamoxifen (catalog #T5648, Sigma-Aldrich, 0.25 mg/kg body weight, typically 4 mg per adult mouse) in peanut oil once per day for four consecutive days by intraperitoneal injection. Tamoxifen administrations were initiated in mice at two and half months of age (P75). Mice were euthanized 4 d or three weeks after the final tamoxifen administration to visualize the cells in which TH is expressed in the GG (4 d) and taste buds (three weeks).

### RNA extraction and real-time RT-qPCR

Total RNA was extracted from GG and TG in wild-type C57BL6/J mice using the RNeasy plus micro kit (QIAGEN, catalog #74034) according to manufacturer instructions. Following isolation, RNA quality was estimated by the RNA Integrity Number (RIN) using a 2100 Bioanalyzer Instrument (Agilent Genomics). Only RNA samples with RIN ≥ 8.0 were used. Both GG and TG cDNA were synthesized from total RNA using Superscript III First-Strand Synthesis SuperMix (Invitrogen). Real-time qPCR was performed using a 7900HT Fast Real-Time PCR System (Applied Biosystems) with FastStart Universal SYBR Green Master Mix (Roche) and oligonucleotide primer sets (Table 1), which were designed from sequences available in the GenBank Database. For each sample, each assay was conducted in three technical replications and the resulting CTs were averaged. For normalization of cDNA loading, all samples were run in parallel with the housekeeping gene *Actin*.

**Table 1:** Sequences of primer pairs used for real-time RT-PCR

Gene GenBank accession #	Sequence 5′−3′	Product size (bp)
*β-Actin* (NM_007393)		154
Forward primer	GGCTGTATTCCCCTCCATCG	
Reverse primer	CCAGTTGGTAACAATGCCATGT	
*TH* (NM_009377.2)		100
Forward primer	CCATCCGGGCTTCTCTGACC	
Reverse primer	TTCCACGTGGGGAATTGGCT	

### Immunofluorescence labeling

Mice were euthanized and transcardially perfused with 4% paraformaldehyde (PFA). Tissues were isolated and then postfixed by immersion in 4% PFA for 2 h or overnight. The tissues were transferred to 30% sucrose and maintained at 4°C for 24 h, frozen in OCT, and stored at −80°C until sectioned on a cryostat. To visualize *Th* expression, GG, tongues, nerves and brain stems were serially sectioned on a cryostat (CM1950; Leica Biosystems) at either 20 or 50 μm and mounted onto precleaned slides (Superfrost plus, ThermoFisher). Immunofluorescence labeling of taste buds in soft palate and nasoincisor ducts was performed with free floating staining of these regions. Cryostat sections were washed by PBS for 15 min at room temperature and then blocked with 10% normal donkey serum (Jackson ImmunoResearch) in PBS containing 0.3% Triton X-100, 1% BSA (Sigma) and mouse-on-mouse blocking reagent (Vector Laboratories). The slides were then incubated with the following primary antibodies, which were diluted in PBS containing 0.3% Triton X-100 with 1% BSA in a humidified chamber overnight, or for 5 d (free floating tissues) at 4°C: rat α–cytokeratin-8 (TROMA-I, supernatant; 1:100 dilution, DSHB], rabbit α-RFP (1:200 dilution; Rockland, catalog #600-401-379), chicken α-RFP (1:200 dilution; Rockland, catalog #600-401-901), goat α-PHOX2B (1:200 dilution; R&D Systems, catalog #AF4940,), mouse α-TUJ1 (βIII-tubulin; 1:200 dilution; Sigma, catalog #T8578), goat α-CAR4 (1:200 dilution; R&D Systems, catalog #AF2414), and rabbit α-TRPM5 (1:200 dilution; Emily Liman’s Lab, University of Southern California). After incubation in primary antibodies, the sections were washed four times in PBS containing 0.3% Triton X-100, and then incubated with secondary antibodies for 4 h or 2 d (free floating tissues) at room temperature (1:200 dilution; Biotinium, donkey antibodies, catalog #CF488, #CF543, or #CF633). Lastly, the tissues were washed three times in PBS with 0.3% Triton X-100 and one time in PBS alone, then mounted with DAPI Fluoromount-G (Southern Biotech).

### *In situ* hybridization

GG sections (20-μm thickness) were washed in PBS for 5 min and then baked at 60°C in an oven for 45 min. After this the sections were fixed with 4% PFA for 1 h at room temperature and then dehydrated by incubating in 50%, 70%, and two times in 100% ethanol for 5 min each. Slides were dried for 5 min at room temperature and incubated for 10 min with RNAScope hydrogen peroxide. Tissue sections were soaked in 95°C distilled water for 10 s and then antigen retrieved at 95°C in RNAScope Target Retrieval solution (Advanced Cell Diagnostics; ACD) for 5 min. Slides were subsequently baked again at 60°C for 45 min. The slides were then subjected to the fluorescence *in situ* hybridization protocol using the RNAScope Multiplex Fluorescent Reagent kit v2 according to the manufacturer guidelines. The following probes were used for all experiments (RNAscope, ACD): *Ret* (431791-C1), *Phox2b* (407868-C2), and *Th* (317621-C3).

### Fluorescence imaging

Immunoreacted GG and taste buds were imaged with a SP8 Lightning confocal microscope (Leica Microsystems) using LAS-X software. Z-stack optical images were captured using a step size of 1 μm at either 20× or 63× magnification with high resolution (2048 × 2048), or were captured using the Lightning Super-resolution settings (Leica Microsystems) at 63× magnification with a 0.5-μm step size. For all images, one PMT channel and two HyD detectors were used to separately capture each fluorophore to generate a composite image.

### Quantification of GG neurons and taste bud cells

For fluorescence *in situ* hybridization experiments, the number of single labeled (*Phox2b*+, *Th*+, *Ret*+) and double-labeled (*Phox2b*+ and *Th*+, *Ret*+ and *Th*+) neurons were counted through each Z-stack section such that neurons were not counted more than once. To distinguish between neurons that expressed high levels of *Th* from those that expressed low levels, images were imported into ImageJ and quantified using the Fiji plugin. The number of pixels were measured in the optical section containing the largest somal diameter for each neuron. Neurons with >100 pixels were considered high *Th* expressors (most had 200–300 pixels), and neurons with <100 pixels were categorized as low *Th* expressors (most low *Th* neurons had less than 50 pixels). For immunolabeling experiments, the number of single labeled (TUJ1+, PHOX2B–, RFP+) and double-labeled (PHOX2B+ and RFP+) neurons were counted through each Z-stack section. The serial sections containing entire GG were counted through each section and added together to determine the total number of neurons in each ganglion. Because fungiform, circumvallate and foliate papillae were serially sectioned (50 μm each section), images of entire taste buds were captured in one or two sections. The total number of taste buds (identified by K8 labeling), taste bud cells (RFP+) and taste buds containing RFP+ fibers were counted by evaluating entire papillae captured on serial sections.

### Statistical analyses

**Figure 1.**
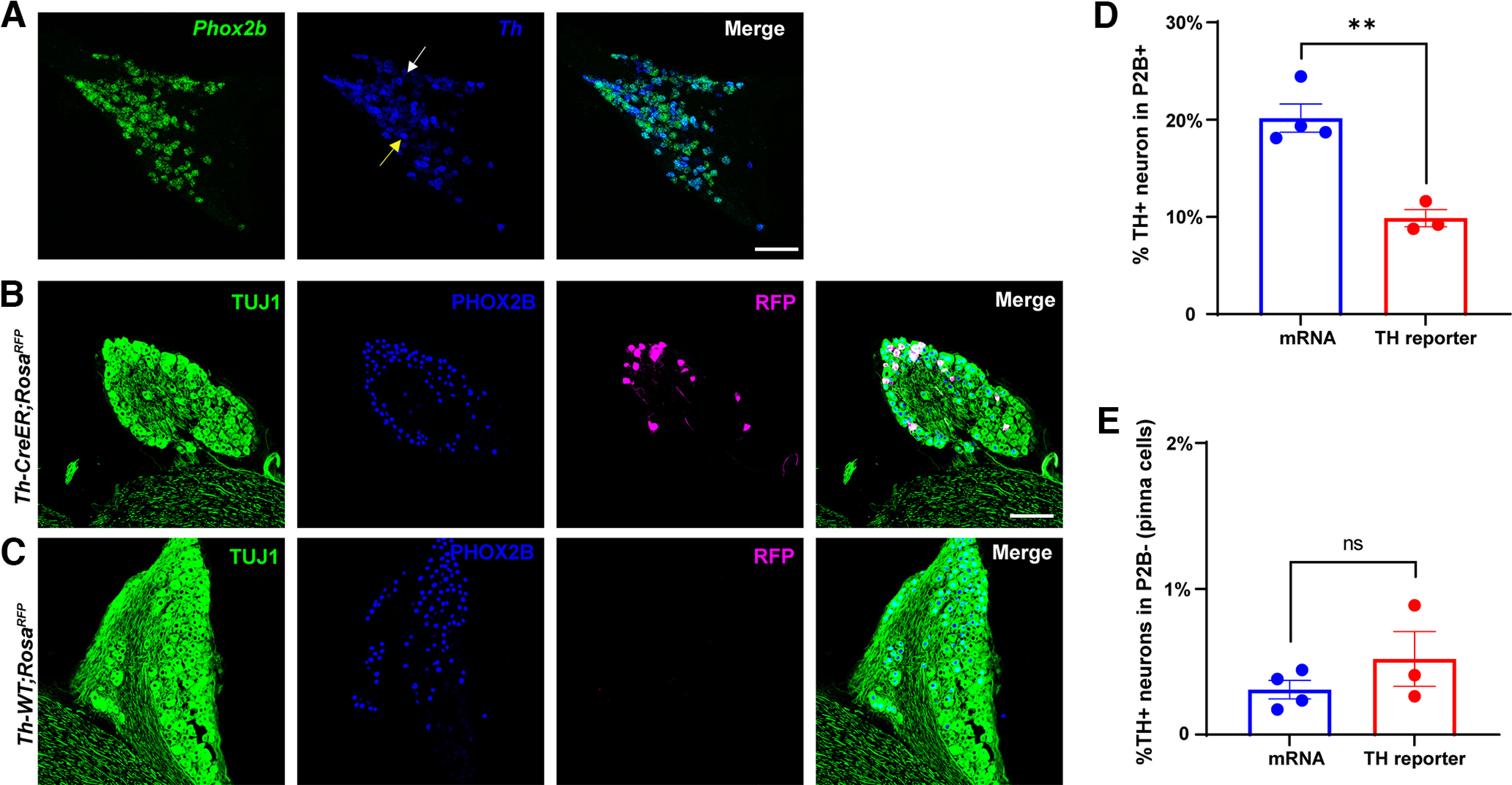
TH expression in the GG. ***A***, Fluorescence *in situ* hybridization labeling of GG sections from adult C57Bl/6 mice using probes to *Phox2b* (green) and *Th* (blue) revealed neurons that express high levels of *Th* (yellow arrow) and low levels of *Th* (white arrow). ***B***, Immunofluorescence labeling of adult GG from *Th*-CreER; *Rosa*^RFP^ mice using antibodies to TUJ1 (green), PHOX2B (blue), and RFP (magenta) identifies a population of PHOX2B+ neurons that also express RFP (*Th*). ***C***, Immunofluorescence labeling of *Th*-WT; *Rosa*^RFP^ mice, as in ***B***, confirms the specificity of the RFP immunoreactions. Immunofluorescence labeling of nodose/petrosal/jugular ganglion complexes from *Th*-CreER; *Rosa*^RFP^ mice using the same antibodies in ***B*** demonstrated the presence of *Th*+/PHOX2B+ oral sensory neurons in petrosal ganglia as well, shown in Extended Data Figure 1-1. ***D***, Serial sections of GG evaluated with both methods were counted and quantified to determine what percentage of *Phox2b*+ neurons coexpressed *Th*. Only high *Th*-expressing neurons are graphed in ***D*** for the *in situ* hybridization. The *p* value is *p* = 0.0027. ***E***, Serial sections of GG were quantified to determine the number of *Th*-expressing neurons that did not express *Phox2b*. Error bars represent mean ± SEM, and *n* = 3–4 mice were analyzed by each method. The *p* value is *p* = 0.2797. Scale bar in ***A*** is 100 μm, and the scale bar in ***B*** is 100 μm and applies to all panels in ***C***. ** denotes *p* < 0.01, and ns is not statistically significant.

**Figure 2.**
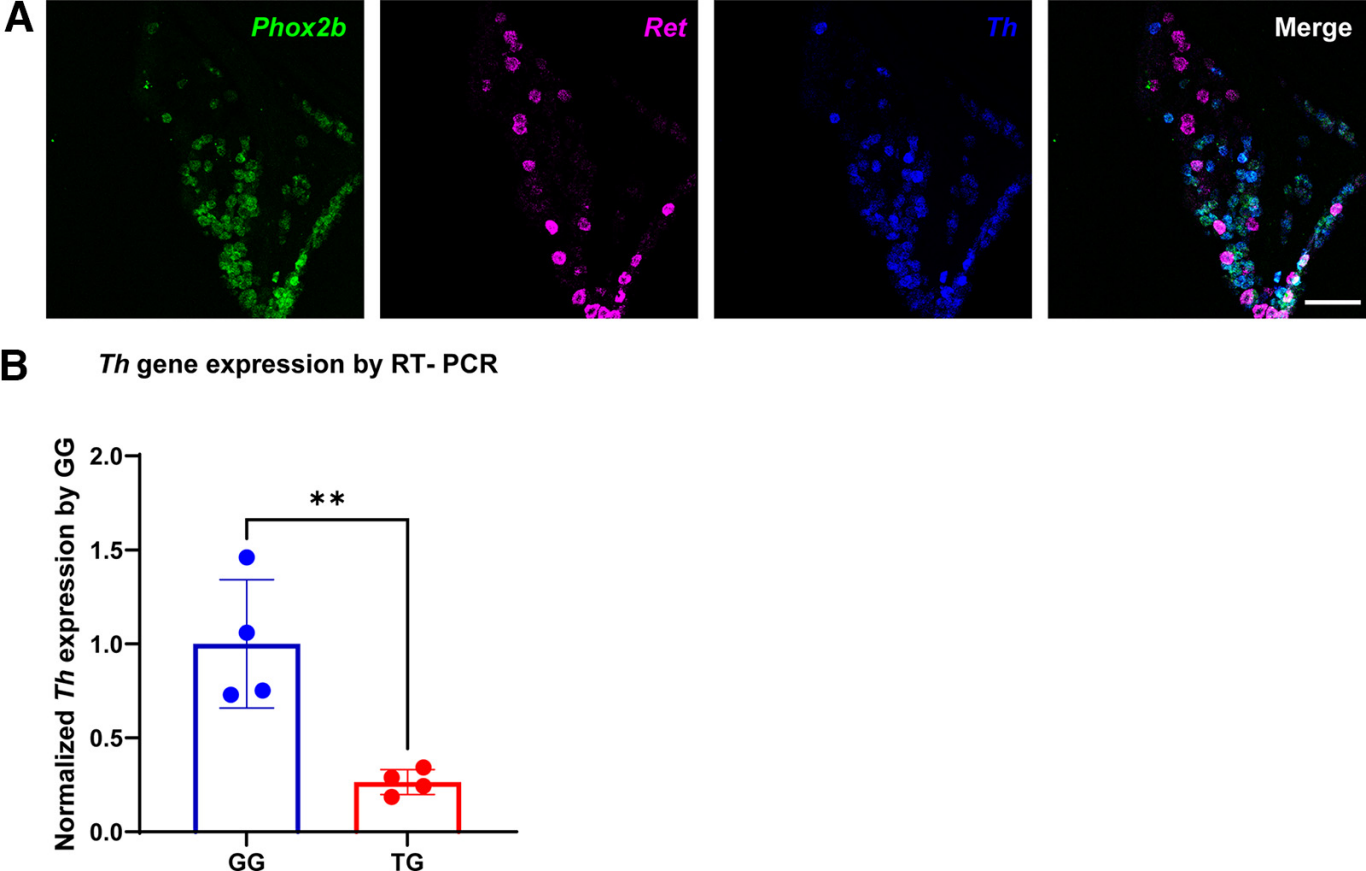
*Th*-expressing geniculate neurons do not express *Ret*. ***A***, Fluorescence *in situ* hybridization was used to examine GG from adult mice with probes to *Phox2b* (green), *Ret* (magenta), and *Th* (blue). *Ret* expression does not overlap with *Th* expression in *Phox2b*+ oral sensory neurons. ***B***, Total RNA isolated from GG and TG were evaluated by RT-qPCR using primers to *Th* and *Actin*. *Th* expression, relative to *Actin*, is higher in the GG than the TG. Error bars are mean ± SEM. Sample sizes were *n* = 3–4 mice and *p* = 0.0055 (** indicates *p* < 0.01). Scale bar: 100 μm.

Quantifications are expressed as mean ± SEM, and all statistical analyses were performed using Prism 8 software (GraphPad). Unpaired Student’s *t* test was used for comparisons of two conditions (comparisons in Figs. 1, 2, and 4). *P* values are represented in all figures as follows: *p < 0.05, **p < 0.01, ***p < 0.001. Sample sizes for each individual experiment are included in the Figure Legends.

10.1523/ENEURO.0523-21.2022.f1-1 Extended Data Figure 1-1 *Th* expression in the nodose/petrosal/jugular ganglion complex. *Th*-CreER; *Rosa*^RFP^ mice were administered tamoxifen and allowed to recover for three weeks. The nodose/petrosal/jugular complex was dissected and sectioned. The sections were immunolabeled with antibodies to TUJ1 (green), PHOX2B (blue), and RFP (cyan). The nodose and petrosal ganglia are often fused together in mice, and the leftmost lobe is the nodose and the rightmost is the petrosal. The jugular ganglion is just anterior along the vagus and is positioned in the lower half of the image, indicated by the white arrow. Some of the PHOX2B+ neurons in nodose/petrosal ganglia are also *Th*+, consistent with innervation of CV tastebuds by *Th*-expressing neurons. Similar results were observed in three mice of each genotype. Scale bar: 100 μm. Download Figure 1-1, TIF file.

## Results

### TH-expressing neurons of the GG are oral sensory neurons that preferentially innervate taste buds on the soft palate

To evaluate whether neurons of the GG express *Th*, we used two complementary methods. The expression of *Th* mRNA was evaluated by fluorescence *in situ* hybridization using a sensitive method capable of detecting very low levels of mRNA (RNAscope, ACD). GG of adult C57Bl/6 mice were serially sectioned and *Th*, along with *Phox2b*, were labeled. *Phox2b* is a transcription factor that is specifically expressed in GG neurons that project to the oral cavity, and not GG somatosensory neurons that project to the pinna, making it a faithful marker of oral sensory neurons (Ohman-Gault et al., 2017). Two populations of *Th*-expressing neurons were identified, neurons that expressed a high level of *Th* (Fig. 1*A*, yellow arrow), and a somewhat larger population that expressed very low levels of *Th* (Fig. 1*A*, white arrow). Nearly all *Th*+ neurons, regardless of the *Th* level expressed, were also *Phox2b+*, indicating they were oral sensory neurons that project to the mouth. The high-expressing *Th+* neurons accounted for 20.1 ± 1.25% of *Phox2b+* neurons and the low-expressing *Th* neurons accounted for an additional 35% of *Phox2b+* neurons (Fig. 1*D*; for quantifications, see Materials and Methods). Our prior experience with fluorescence *in situ* hybridization, using the RNAscope method that is sensitive enough to detect a single transcript, is that low expression of mRNA does not result in protein expression. Therefore, as a second method for the evaluation of *Th* expression, a transgenic mouse expressing CreER under the *Th* promotor (*Th*-CreER; Abraira et al., 2017) was crossed with a TdTomato reporter line (The Jackson Laboratory, stock #007914) such that *Th*-expressing neurons would be labeled with the fluorescent tdTomato protein (RFP) after tamoxifen administration. Tamoxifen administration of adult *Th*-CreER; *Rosa*^RFP^ mice followed by immunofluorescence labeling of RFP revealed a small population of geniculate neurons that were highly RFP+ (Fig. 1*B*). Quantification of this population determined that 9.87 ± 0.88% of PHOX2B+ geniculate neurons expressed *Th* (RFP) via this reporter (Fig. 1*D*) and <1% of PHOX2B– neurons expressed *Th* (Fig. 1*E*). RFP immunolabeling of tamoxifen-treated *Th*-WT; *Rosa*^RFP^ mice did not result in any RFP labeling, confirming that RFP immunoreactions were specific for neurons expressing *Th* (Fig. 1*C*). It should be noted that a commercially available *Th*-Cre line (The Jackson Laboratory, stock #008601; Savitt et al., 2005) showed very little labeling in both the GG and the superior cervical ganglion, which is known to be composed of nearly 100% TH-expressing neurons, suggesting that Cre recombination in peripheral ganglia in this *Th*-Cre line was low. Taken together, these data suggest that neurons expressing a high level of *Th* mRNA (20%) most likely encompass the 10% of oral sensory neurons that are RFP+ and, therefore, express *Th* using a reporter line. Furthermore, *Th* was nearly absent from PHOX2B-negative, pinna-projecting sensory neurons.

In dorsal root ganglia (DRG) 15–30% of neurons express TH, depending on their vertebral level, and distinguish c-fiber low threshold mechanoreceptors (c-LTMRs) and small diameter neurons that innervate blood vessels and regulate blood pressure (Price and Mudge, 1983; Brumovsky et al., 2006, 2012; Li et al., 2011; Morelli et al., 2021). Because c-LTMRs respond to light, affective touch, we wondered whether recently-identified *Ret*-expressing mechanoreceptors in the GG (Donnelly et al., 2018) could be c-LTMRs and communicate pleasurable or aversive food textures. In the GG, there are two groups of *Ret*-expressing neurons: PHOX2B+, *Ret*-expressing neurons are large-diameter mechanosensory neurons, and PHOX2B–, Brn3A+, *Ret*-expressing neurons are a subgroup of smaller diameter pinna-projecting neurons (Donnelly et al., 2018). We explored whether *Th*+ geniculate neurons also expressed *Ret*. Using fluorescence *in situ* hybridization probes for *Th*, *Ret* and *Phox2b,* we determined that high-expressing *Th*+ neurons that are oral sensory neurons (*Phox2b+*) did not express *Ret* (Fig. 2*A*). In fact, *Th+* and *Ret+* neurons were largely mutually exclusive in the GG, suggesting that *Th*+ oral sensory neurons are unlikely to be mechanosensory neurons.

Many of the somatosensory neurons of the TG innervate the oral cavity, including the extragemmal regions of taste papillae. To compare the extent to which TG neurons express *Th*, as compared with geniculate neurons, quantitative reverse transcription-PCR (RT-qPCR) was performed on RNA isolated from TG and GG. These ganglia were dissected from adult C57Bl/6 mice, total RNA isolated from them, and RT-qPCR was performed using primers to *Th* and *Actin*. Interestingly, *Th* was expressed at a significantly higher level in GG as compared with TG, when standardized to *Actin* levels (Fig. 2*B*).

**Figure 3.**
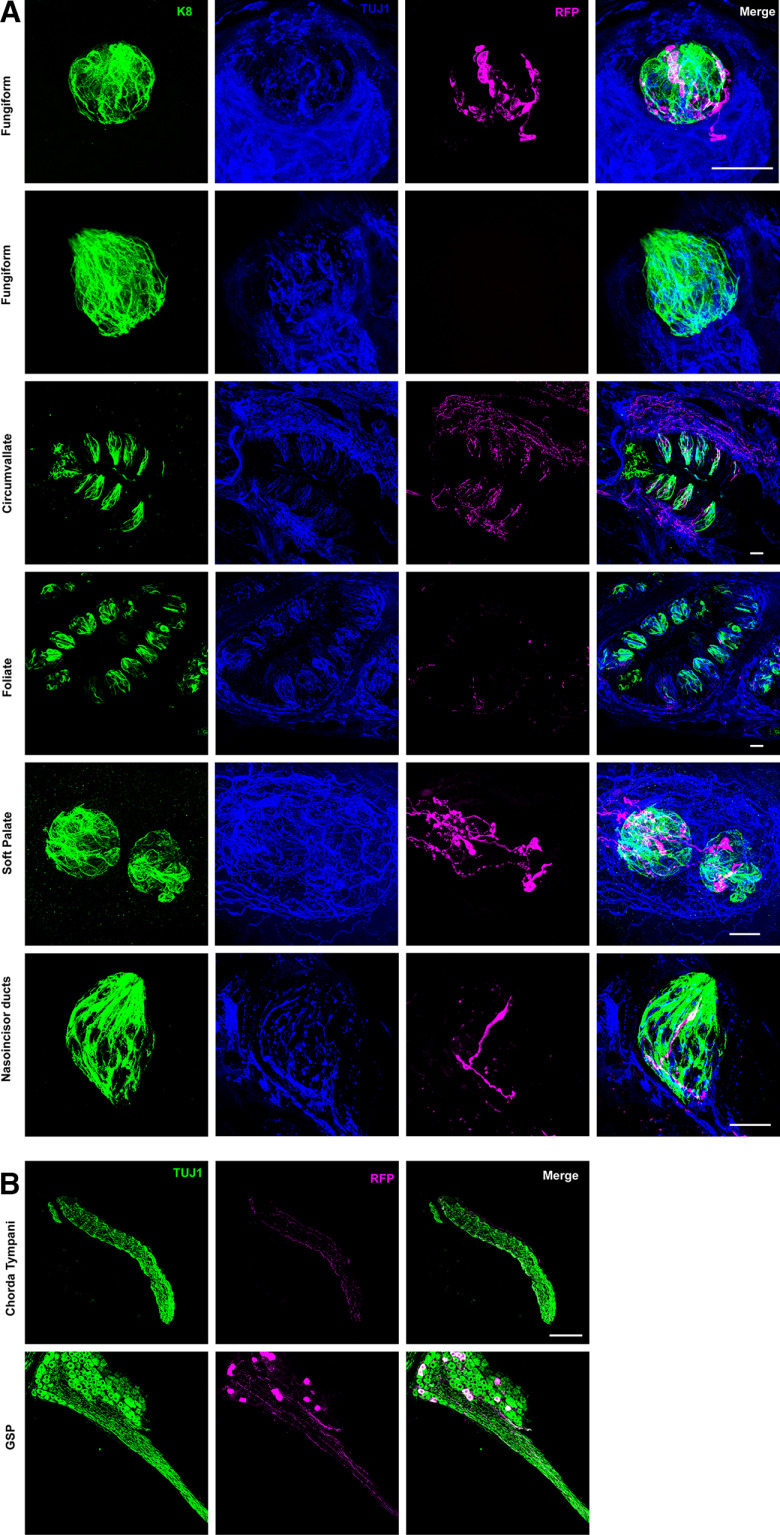
Fibers from *Th*-expressing neurons preferentially innervate taste buds in the soft palate and circumvallate papillae. ***A***, Fungiform (top two rows), circumvallate (third row), anterior foliate (fourth row), soft palate (fifth row), and nasoincisor duct (sixth row) taste buds were immunolabeled with antibodies to K8 (green), TUJ1 (blue), and RFP (magenta). RFP+ fibers innervated half of fungiform, anterior foliate and nasoincisor duct taste buds, and innervated all circumvallate and soft palate taste buds. For fungiform taste buds, images are displayed in two separate rows depicting an example of innervated (top) and noninnervated (second row) taste buds. ***B***, Chorda tympani and greater superficial petrosal (GSP) nerves were immunofluorescently labeled with antibodies to TUJ1 (green) and RFP (magenta), showing the presence of RFP+ fibers projecting through each nerve. Three mice were imaged in these experiments. Scale bars in ***A*** are 20 μm, and the scale bar in the top row of fungiform taste bud images applies to the second row as well. The scale bar in ***B*** is 100 μm and applies to all panels in ***B***.

To determine the innervation patterns of TH-expressing oral sensory neurons, we performed RFP immunofluorescence labeling on the tongues and palates of *Th*-CreER; *Rosa*^RFP^ mice. Adult mice were administered tamoxifen, and three weeks after the last injection, they were euthanized and tissues were collected. Analysis of fungiform taste buds revealed two patterns of innervation: taste buds had either no innervation from RFP+ fibers, or they were robustly innervated by RFP+ fibers (Fig. 3*A*). Regardless of whether there was taste bud innervation, we did not observe extragemmal RFP+ fibers in fungiform papillae, and all RFP+ fibers coursed directly into taste buds (Fig. 3*A*). Anterior foliate taste buds, which like fungiform taste buds are also innervated by fibers from the chorda tympani, had a similar pattern of innervation in that many taste buds were not innervated, while others were thoroughly innervated (Fig. 3*A*). Quantification of the number of taste buds that are innervated by RFP+ fibers indicated that 49.65 ± 1.36% of fungiform taste buds were innervated (47–53 taste buds analyzed in each mouse) and 39.01 ± 2.04% of anterior foliate taste buds were innervated (57–65 taste buds analyzed in each tongue), with the remaining ∼50% and 60% of taste buds, respectively, being vacant. While GG innervation of fungiform and anterior foliate taste buds is predominantly derived from the chorda tympani nerve, the greater superficial petrosal nerve, which also emanates from the GG, innervates taste buds located on the soft palate in the posterior oral cavity and taste buds in the nasoincisor ducts of the anterior oral cavity (Cleaton-Jones, 1976; Miller et al., 1978; Miller and Spangler, 1982). Upon evaluation we observed that 100% of observed soft palate taste buds displayed robust innervation by RFP+ fibers (Fig. 3*A*, 25–30 taste buds in each mouse), in contrast to fungiform and anterior foliate taste buds. Similar to other anterior regions, only 25% of taste buds in the nasoincisor ducts were innervated, and those that were innervated only contained one to two RFP+ fibers (Fig. 3*A*, 20 taste buds analyzed in each mouse). Consistent with these observations, both the chorda tympani and greater superficial petrosal nerves had RFP+ fibers from *Th*+ neurons projecting through them, with the density of fibers appearing somewhat higher in the greater superficial petrosal nerve (Fig. 3*B*).

Circumvallate papillae (CV), which are innervated by oral sensory neurons of the petrosal ganglion, were heavily innervated by *Th*+ neurons (Fig. 3*A*), and quantification indicated that all observed CV taste buds were innervated by RFP+ fibers (99–110 taste buds analyzed in each mouse). Because of this innervation pattern, we evaluated *Th* expression in petrosal neurons. In rodents the petrosal ganglion is part of the nodose-petrosal-jugular complex that is located on the vagus nerve near the bifurcation of the carotid artery. Nodose-petrosal-jugular complexes from *Th*-CreER; *Rosa*^RFP^ mice were isolated and immunolabeled for PHOX2B, TUJ1, and RFP. A subset of nodose-petrosal neurons, all of which express PHOX2B, were also RFP+, as expected (Extended Data Fig. 1-1; Prescott et al., 2020). The jugular ganglion, located just rostral to the nodose-petrosal ganglion on the vagus nerve, is derived from the neural crest and therefore does not express PHOX2B (Prescott et al., 2020). These neurons did not express RFP, indicating that jugular ganglion neurons are not likely to express *Th* (Extended Data Fig. 1-1). Taken together, geniculate oral sensory neurons that express *Th* preferentially innervate taste buds in the posterior tongue and soft palate than taste buds in more anterior regions of the tongue, i.e., the fungiform papillae, anterior foliate papillae, and nasoincisor ducts.

### TH expression marks a rare Type II taste bud receptor cell

**Figure 4.**
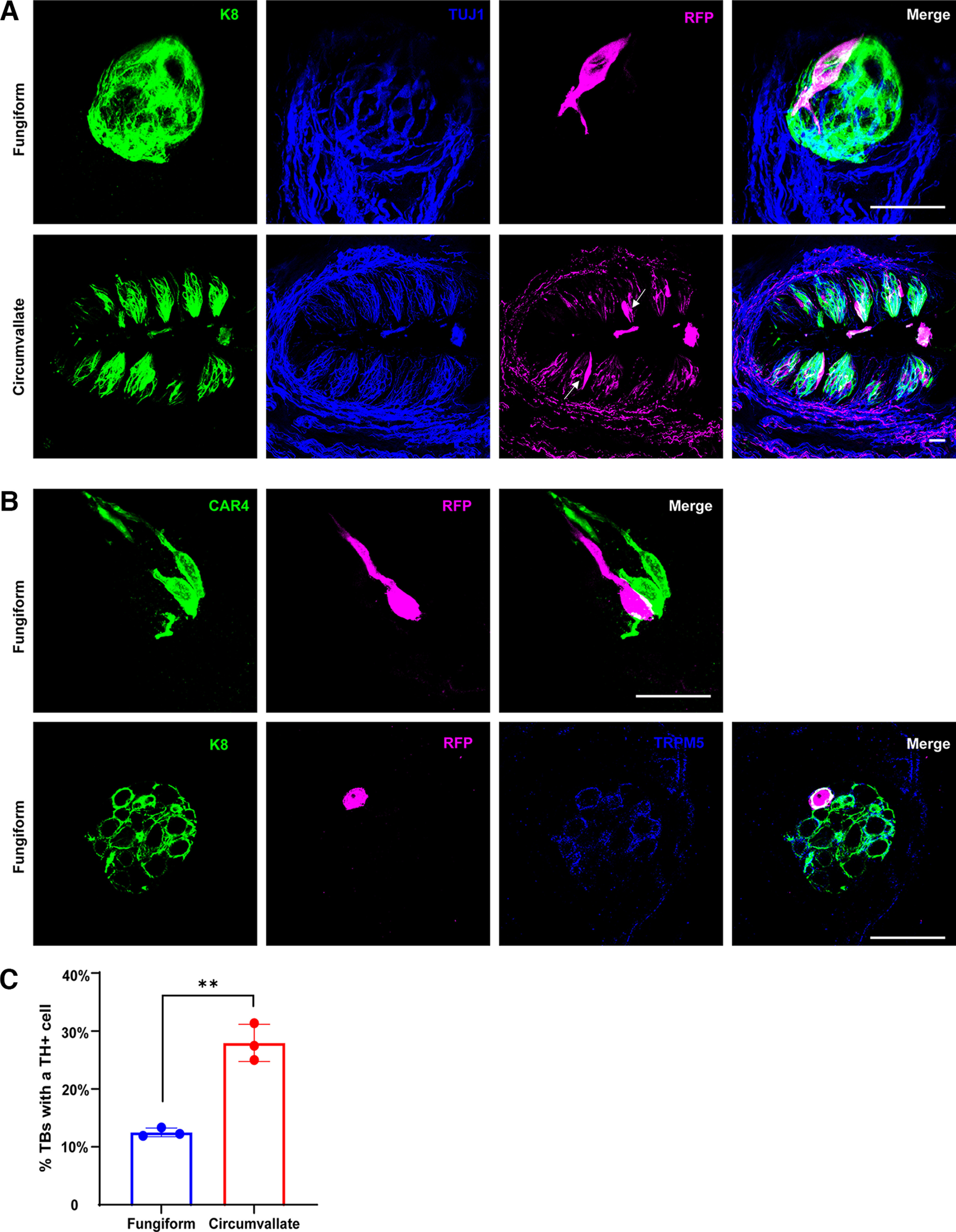
*Th*-expressing taste bud cells are a rare population of TRPM5+ Type II receptor cells. ***A***, Fungiform (top row) or circumvallate (second row) taste buds from *Th*-CreER; *Rosa*^RFP^ mice were immunolabeled for K8 (green), TUJ1 (blue), and RFP (magenta). Tissues were collected 3–4 d after the last tamoxifen injection. ***B***, Fungiform taste buds from tamoxifen-treated *Th*-CreER; *Rosa*^RFP^ mice were immunofluorescently labeled with antibodies to CAR4 (green) and RFP (magenta) or with antibodies to K8 (blue), RFP (magenta), and TRPM5 (blue). RFP+ cells did not overlap with CAR4 labeling (top row in ***B***) but did overlap with TRPM5 labeling (bottom row). ***C***, Quantifications of the number of fungiform and circumvallate taste buds containing a RFP+ taste receptor cell. For fungiform taste buds, 42–49 taste buds were analyzed in each of three mice; for circumvallate taste buds 96–118 taste buds were evaluated each from three mice. Data are graphed as the mean ± SEM, *n* = 3, and *p* = 0.0012. Scale bars are 20 μm and ** indicates *p* < 0.01.

On occasion *Th*-CreER; *Rosa*^RFP^ mice were administered tamoxifen and then euthanized shortly after the last injection, within 4 d. When innervation of the tongue was examined using RFP immunolabeling, we observed highly RFP (*Th*)*+* taste bud cells in both fungiform and circumvallate papillae (Fig. 4*A*). There was never more than one RFP+ cell per taste bud, and only a few taste buds contained these cells. The number of taste buds containing RFP+ receptor cells was quantified, and we observed that only 12% of fungiform taste buds, and 28% of circumvallate taste buds, contained a RFP+ cell (Fig. 4*C*). If we waited for three weeks after the last tamoxifen injection to euthanize the *Th*-CreER; *Rosa*^RFP^ mice and perform RFP immunolabeling, no RFP+ cells were observed in any taste bud, indicating that these cells turn over within three weeks. To determine whether these cells were Type II or Type III cells, we used markers specific for these cell classes. CAR4 labels most Type III cells (Chandrashekar et al., 2009; Lossow et al., 2017; Wilson et al., 2017), and CAR4 immunolabeling of taste buds from *Th*-CreER; *Rosa*^RFP^ mice that were colabeled with RFP demonstrated that these *Th*-expressing cells did not express CAR4, and were therefore unlikely to be Type III cells (Fig. 4*B*). Interestingly, immunolabelling taste buds from *Th*-CreER; *Rosa*^RFP^ mice with TRPM5 antibodies along with RFP antibodies revealed that these *Th*+ cells were colabeled with TRPM5, indicating that they are a Type II receptor cell (Dutta Banik et al., 2018; Z Zhang et al., 2007; Fig. 4*B*). Therefore, *Th*-expressing taste bud cells are an uncommon Type II cell that turns over within three weeks.

### The central projections of TH-expressing oral sensory neurons innervate the rostral nucleus of the solitary tract (NTS)

**Figure 5.**
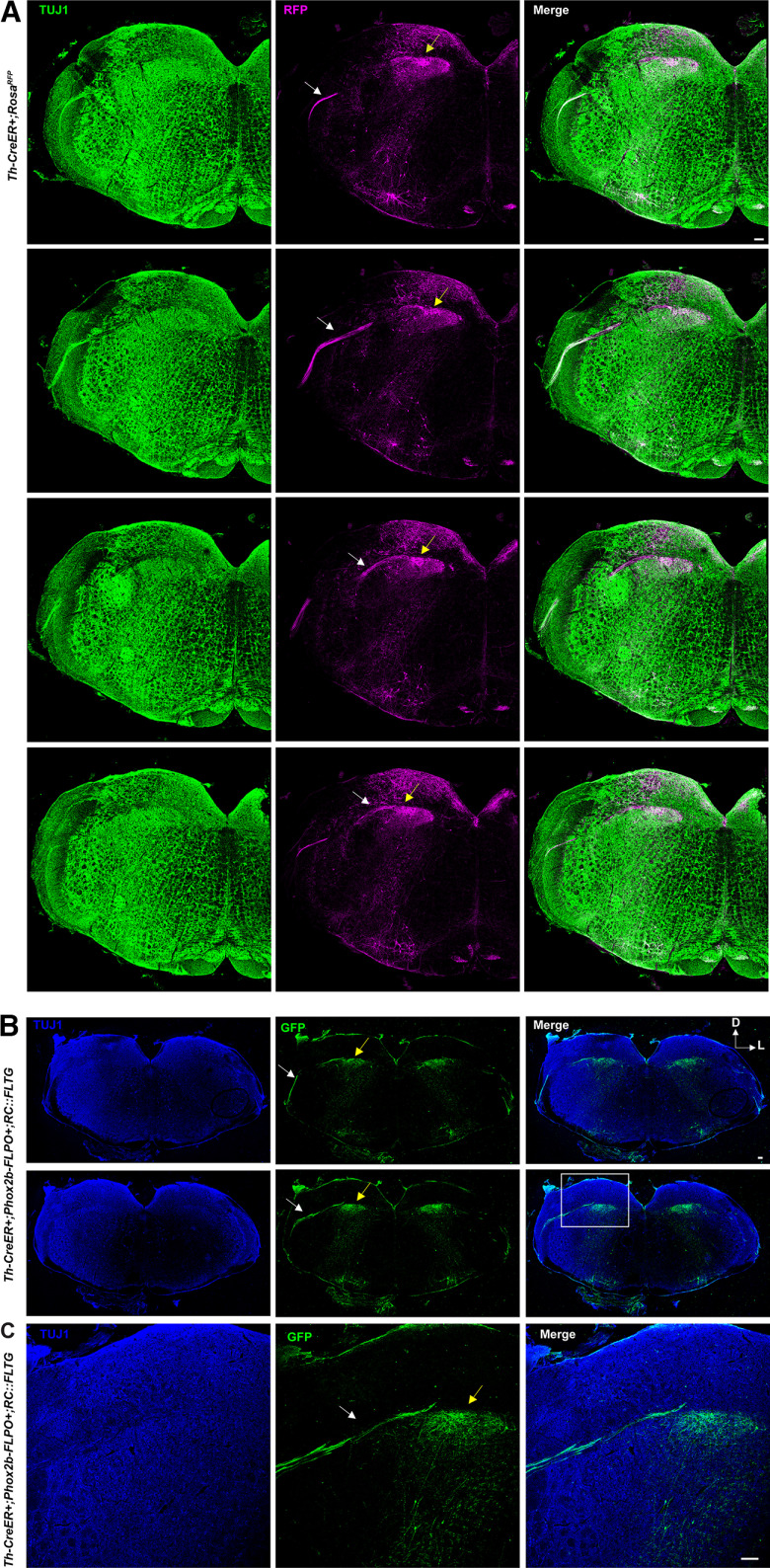
Fibers from *Th*+ oral sensory neurons enter the rostral portion of the NTS. ***A***, *Th*-CreER; *Rosa*^RFP^ mice were administered tamoxifen and allowed to recover for three weeks before the brainstem was isolated. Coronal serial sections through the NTS were immunolabeled with antibodies to TUJ1 (green) and RFP (magenta). The white arrows indicate the solitary tract that is lateral to the NTS (indicated with yellow arrows). Successive sections are displayed progressing from top row to bottom row so that the progression of the solitary tract to the NTS can be appreciated. Scale bar: 100 μm. ***B***, *Th*-CreER; *Phox2b*-FLPO; RC::FLTG mice were administered tamoxifen and allowed to recover for three weeks. Coronal serial sections were immunolabeled with antibodies to TUJ1 (blue) and eGFP (green). The white arrows indicate the solitary tract and yellow arrows denote the NTS. The bottom row is a higher power image of the row above it. The scale bars are 100 μm. The scale bar in the first row in ***A*** applies to all panels in ***A***, and the scale bar in ***B*** applies to all panels in ***B***. Four independent mice of each genotype were analyzed in ***A*** and ***B***.

To determine where GG neurons that express *Th* terminate in the NTS, which is the first CNS nucleus innervated by oral sensory neurons, we performed RFP immunofluorescence labeling of *Th*-CreER; *Rosa*^RFP^ mice three weeks after tamoxifen injection. Serial coronal sections through the NTS were immunolabeled for RFP and TUJ1, a general neuronal marker. The most rostral region of the NTS was examined because this is the entry zone for chemosensory fibers arriving from cranial nerves VII, IX and X. The solitary tract was clearly visible in serial sections and contained RFP+ fibers (Fig. 5*A*, white arrow indicates solitary tract). RFP+ fibers were also observed entering the rostral NTS, suggesting that *Th*-expressing oral sensory neurons project into the gustatory region of the NTS via the solitary tract, as expected (Fig. 5*A*, yellow arrow). There were, however, *Th*+ neurons within the NTS itself, as determined by the presence of RFP+ cell bodies, and their processes, in the rostral region (Fig. 5*A*). This made it difficult to fully appreciate the extent to which *Th*+ fibers projected and terminated within the NTS, given the presence of dense RFP+ projections throughout the rostral NTS. To better evaluate these *Th* projections, intersectional genetics was used to label axons from neurons that express both *Phox2b* and *Th* simultaneously using *Th^CreER^* mice and transgenic mice expressing FLP recombinase under the Phox2b promoter (*Phox2b^FLPO^*). Although *Phox2b* is expressed in the NTS during embryonic development, its expression is limited in adulthood (D’Autreaux et al., 2011). Therefore, when tamoxifen was applied to activate Cre in *Th* neurons of adult mice, FLPO is no longer expressed, and these neurons will not be labeled with GFP, the dual-recombinase reporter. Serial coronal sections through the medulla were immunolabeled with TUJ1 to label all neurons, and with GFP to label *Th*+/*Phox2b+*-expressing neurons (Fig. 5*B*). As before, the solitary tract was clearly visible (white arrow) because of the presence of GFP+ axons that entered the rostral NTS (yellow arrow). The intersectional labeling method resulted in a great reduction in the number of GFP+ neurons present in the NTS, making the entry of GFP+ fibers clear. Thus, *Th*-expressing peripheral oral sensory neurons project into the rostral gustatory region of the NTS, as expected.

### Fibers from TH+ oral sensory geniculate neurons contact both Type II and Type III taste bud cells

**Figure 6.**
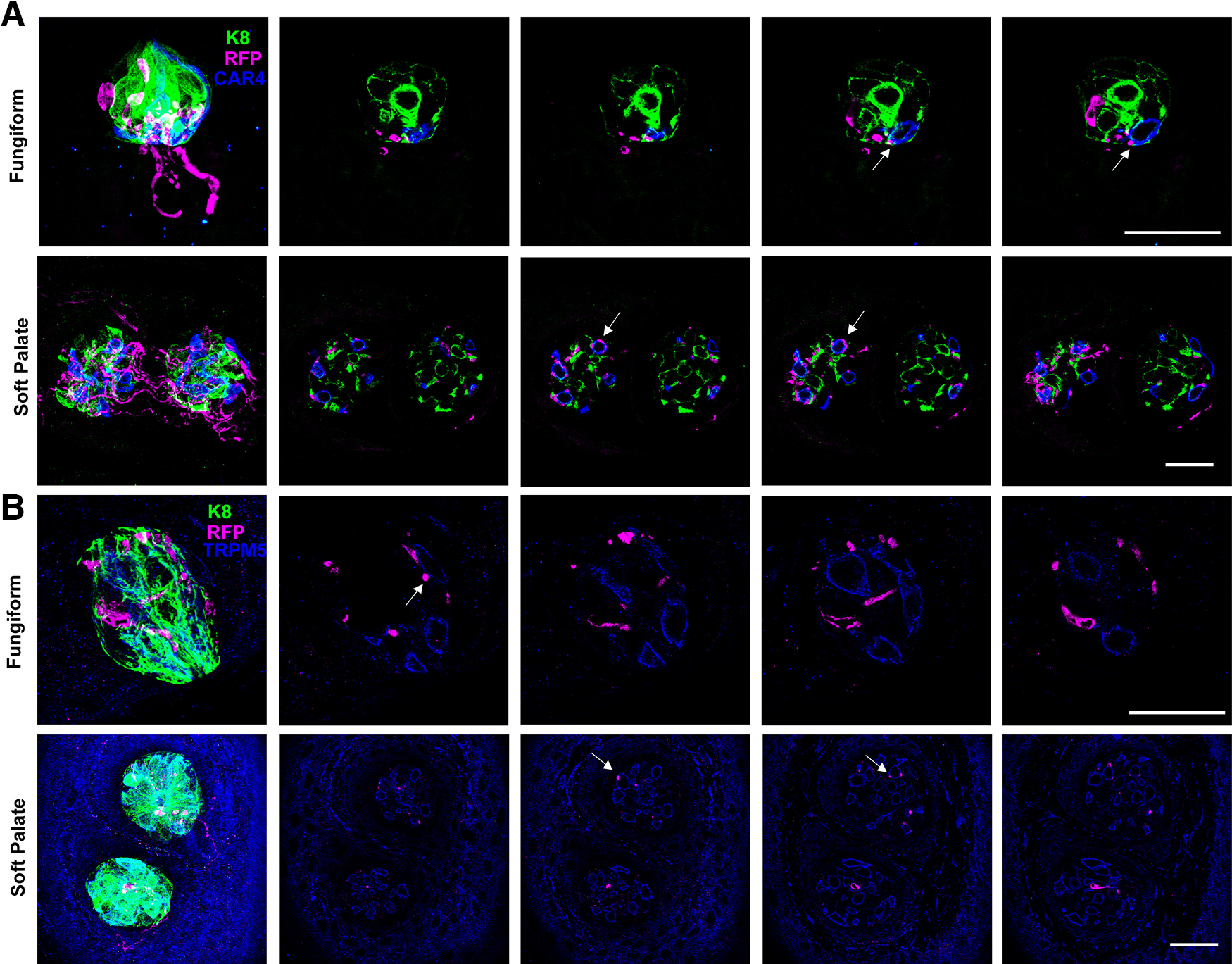
Intragemmal fibers from *Th*-expressing geniculate ganglion neurons contact both Type II and Type III taste bud cells. ***A***, Fungiform taste buds (top row) and soft palate taste buds (second row) were immunolabelled for K8 (green), RFP (magenta) and CAR4 (blue). Maximum projections of 30- to 40-μm Z-stacks are shown on the left, and four individual slices at different levels in the Z-stack are shown to the right. Points of apparent contact between RFP+ fibers and CAR4+ cells are indicated with white arrows. ***B***, K8 (green), RFP (magenta), and TRPM5 (blue) immunolabeling is shown for fungiform (top) and soft palate (bottom) taste buds, similar to ***A***. The four individual slices shown to the right of the maximum projections of the Z-stacks (left-most panel) only display RFP and TRPM5 labeling for clarity. White arrows indicate regions of contact between RFP+ fibers and TRPM5+ taste bud cells. Three mice were independently analyzed in ***A*** and ***B***, and five to seven taste buds were imaged and analyzed in each mouse. Scale bars: 20 μm.

Because *Th+* geniculate neurons innervate taste buds, and not extragemmal regions around taste buds, and since these neurons do not express *Ret*, they are more likely to be chemosensory neurons than somatosensory neurons (Donnelly et al., 2018, 2022). To determine whether *Th*+ fibers appear to contact taste bud cells, immunolabelling experiments with antibodies to Type II and Type III cells were performed on serial sections of taste buds of tamoxifen-treated *Th*-CreER; *Rosa*^RFP^ mice. Sections of fungiform papillae and soft palate were immunolabeled for K8, a general taste bud cell marker, RFP (*Th*+ fibers), and CAR4. RFP-labeled fibers that project into taste buds appeared to contact CAR4+ cells throughout fungiform and soft palate taste buds (Fig. 6*A*). There were some RFP+ fibers, however, that did not appear to contact CAR4+ cells, as well as some CAR4+ cells that were not contacted by RFP+ fibers, although the majority of CAR4+ cells appeared to be contacted (Fig. 6*A*). When immunolabeling was also performed with antibodies to K8, RFP and TRPM5, we observed RFP+ fibers that appeared to contact TRPM5+ taste bud cells as well (Fig. 6*B*). TRPM5+ taste receptor cells were contacted by RFP+ fibers in taste buds present in both fungiform papillae and soft palate. TRPM5+ cells, however, were frequently not contacted by RFP+ fibers, and fewer than half of the TRPM5+ cells we observed had contacts from RFP+ fibers (Fig. 6*B*).

**Figure 7.**
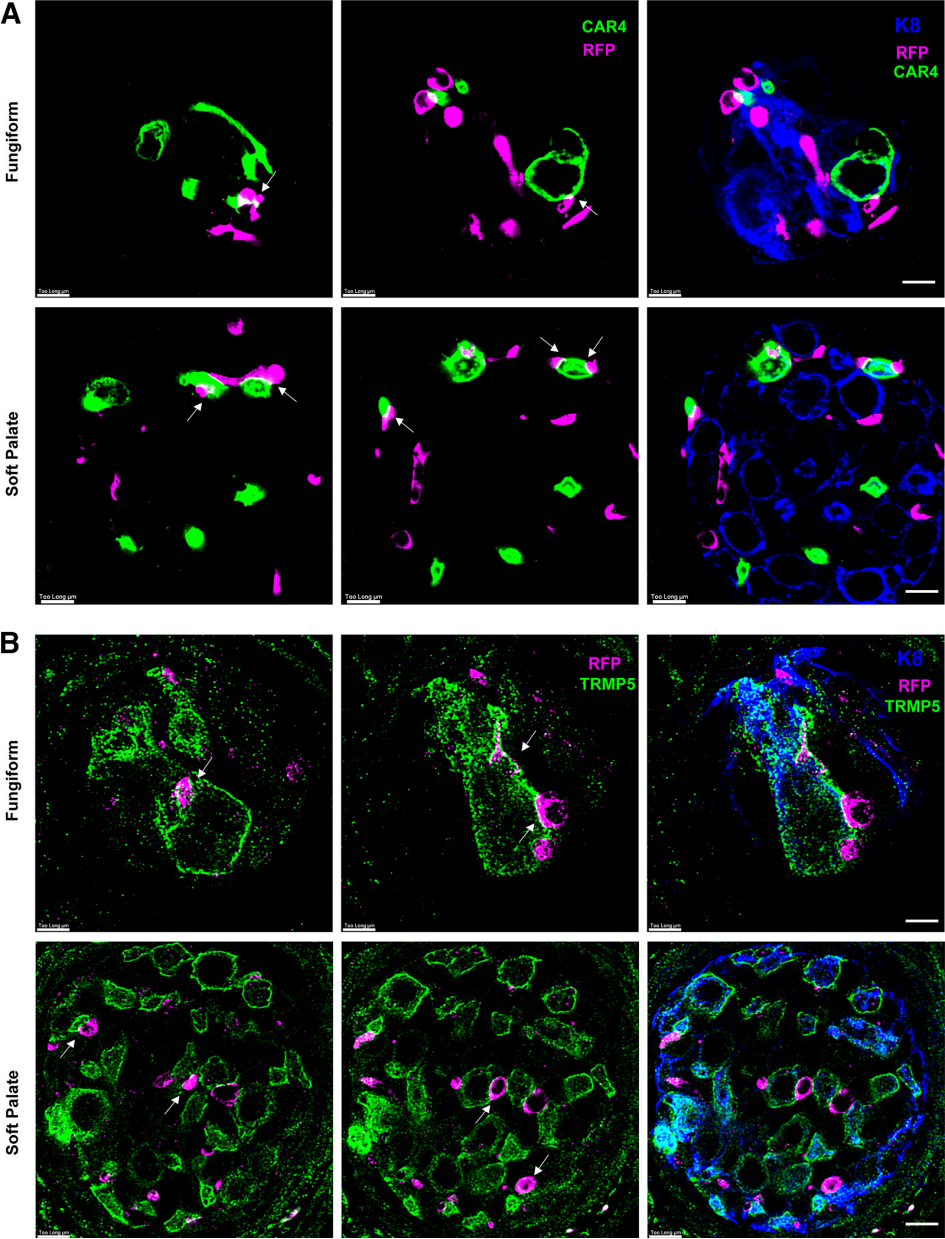
Points of contact between intragemmal fibers from *Th*-expressing neurons and Type II and Type III cells have synapse-like morphology. Immunolabelling for (***A***) CAR4 (green), RFP (magenta), and K8 (blue, in rightmost merged images) or (***B***) TRPM5 (green), RFP (magenta), and K8 (blue, in merged panels) is shown for a representative fungiform taste bud (top row) and a soft palate taste bud (bottom row). Super-resolution confocal imaging revealed that regions of overlap between CAR4 (or TRPM5) and RFP displayed membrane invaginations and flattened axonal regions typical of synaptic contacts, indicated with white arrows. Three to four mice were analyzed for each experiment, and five to seven taste buds were imaged and evaluated in each mouse. Scale bars: 5 μm.

Many of the RFP+ fibers we traced contacted the same CAR4+ or TRPM5+ cell multiple times, or had large regions of contact (Fig. 6). The close proximity between taste bud cells and intragemmal fibers, however, does not necessarily mean a synapse is present between the two cell types in question, and contacts may simply represent intragemmal fibers passing by a taste cell to make a contact elsewhere in the taste bud. To better observe these points of contact between RFP+ fibers and taste bud cells, we performed super-resolution confocal microscopy (SP8 Lightning, Leica) on fungiform and soft palate taste buds from *Th*-CreER; *Rosa*^RFP^ mice. Imaging of RFP-labeled fibers with either CAR4+ (Fig. 7*A*) or TRPM5+ (Fig. 7*B*) taste bud cells revealed that at many of these points of contact, RFP+ fibers caused invaginations within the CAR4+/TRPM5+ cells reminiscent of synapses. In some cases, the immunolabelled taste bud cells partially enveloped the RFP+ axon (Fig. 7, white arrows). We also observed numerous examples of CAR4+/TRPM5+ cell membranes that contacted RFP+ fibers that were flattened and appeared to encompass large local portions of the CAR4+/TRPM5+ cell surface. These types of complex morphologies would not be expected from axons indiscriminately contacting taste bud cells while projecting to the intended target, and are more likely to represent synaptic contacts, including the atypical synapses of Type II cells. In summary, *Th+* oral sensory neurons contact both Type II and Type III taste bud cells, and these contacts often have synapse-like morphologies, raising the possibility that these *Th+* neurons could respond to multiple taste qualities.

## Discussion

Molecular genetic tools allowed for the identification of a small population of GG oral sensory neurons, accounting for 10–20% of PHOX2B+ neurons, depending on the method used, that robustly express *Th*, the rate-limiting enzyme in the production of catecholamines. These neurons innervated fungiform taste buds, anterior foliate taste buds, taste buds in nasoincisor ducts, and taste buds in the soft palate, indicating that they project through the chorda tympani nerve and greater superficial petrosal nerve. *Th*-expressing fibers also innervated circumvallate taste buds, and we observed *Th*+ neurons in *Phox2b*-expressing neurons of the mouse nodose/petrosal/jugular ganglion complex. All of the observed taste buds in the most posterior region of the tongue (circumvallate papilla) and soft palate were innervated, as compared with taste buds more anteriorally located on the tongue (fungiform, anterior foliate, nasoincisor duct) which were only 35–50% innervated. As expected, oral sensory *Th+* neurons project into the rostral NTS, and their peripheral axons contact both CAR4-expressing and TRPM5-expressing taste bud cells in fungiform and soft palate taste buds. In addition to identifying this interesting population of gustatory neurons, *Th* expression also labeled a rare TRPM5+ Type II receptor cell that is found in only a small fraction of fungiform and circumvallate taste buds.

Analysis of innervation patterns of anterior foliate papillae, fungiform papillae and soft palate suggest that TH projections only have intragemmal fibers, and do not project to extragemmal regions outside of taste buds. In fact, *Th*+ fibers were not observed in the nontaste filiform papillae or the surrounding lingual epithelium. In circumvallate papillae, numerous *Th*+ fibers were observed basal to taste buds (Figs. 3, 5), making it difficult to say unequivocally that there were no extragemmal fibers in this region. In fungiform papillae, fibers from oral sensory PHOX2B+ neurons predominantly course into taste buds, but some of these fibers take an extragemmal route to project apical to taste buds, terminating just under the lingual epithelium (Donnelly et al., 2022). These axons are most likely somatosensory in nature, given they persist largely unaltered after treatment with hedgehog pathway inhibitors that eliminate taste buds and chemical responses, but not tactile responses (Kumari et al., 2015, 2017, 2018; Donnelly et al., 2022). This apparent lack of extragemmal *Th*+ fibers, along with the observation that *Th*+ fibers contact TRPM5-expressing and Car4-expressing receptor cells, suggests these neurons are chemosensory. *Th*+ geniculate neurons did not express *Ret*, and given that *Ret*+ neurons that also express PHOX2B in the GG respond to tactile stimulation of the tongue surface (Donnelly et al., 2018), *Th*+ neurons are unlikely to be somatosensory, reinforcing the likelihood they are chemosensory in nature.

Because we observed synapse-like contacts on both TRPM5+ Type II cells and CAR4+ Type III cells, it is tempting to speculate that these neurons respond to more than one taste quality. It is possible, however, that *Th*-expressing neurons are not a single, homogenous population of cells, but are instead two different types of neurons that either innervate Type II cells, or Type III cells, and not both. The number of PHOX2B+ geniculate neurons that robustly express *Th* are between 10% and 20%, depending on the method used, or ∼50–100 neurons per ganglion, assuming there are 500 oral sensory neurons per mouse GG. This suggests that each neuron innervates more than one taste bud to account for the number of taste buds that contain *Th*+ fibers. While many oral sensory geniculate neurons only innervate a single taste bud, recent sparse neuron labeling methods in fungiform papillae revealed that just over 50% of oral sensory neurons innervate more than one taste bud, with some neurons having very complex branching patterns (Zaidi and Whitehead, 2006; T Huang et al., 2021). Interestingly, almost a third of geniculate oral sensory neurons contact both PLCβ2-labeled cells and CAR4-labeled receptor cells (T Huang et al., 2021), allowing for the possibility that *Th+* neurons innervate both cell types and would be likely to respond to more than one taste quality. Ultimately, genetic sparse labeling of *Th*+ neurons followed by analysis of their receptive fields would be the most unequivocal method to address this question.

The observation that only 50% of fungiform and anterior foliate taste buds are innervated by *Th*+ neurons, as opposed to 100% of soft palate taste buds, suggests that a larger percentage of *Th*+ geniculate neurons project through the greater superficial petrosal nerve. All circumvallate taste buds appeared to have innervation by *Th*+ fibers as well. These data raise the possibility that this neuron subtype plays more of a role in taste on the palate and very back of the tongue, as opposed to the more anterior surface of the tongue. Interestingly, this pattern of circumvallate taste buds being innervated more heavily than fungiform taste buds is similar to the abundance of Type III cells, which are more abundant in taste buds in the back of the tongue (Roper and Chaudhari, 2017; Wilson et al., 2017).

The potential role of catecholamines in taste neurotransmission in the periphery is not fully understood. Innervation of taste buds by TH-expressing fibers has been observed in circumvallate papillae, but not fungiform papillae or larynx (Yamamoto et al., 1997; Dvoryanchikov et al., 2007; Ohman-Gault et al., 2017). This is consistent with our observations that all circumvallate taste buds are innervated, whereas only half of fungiform taste buds are innervated (Fig. 3). Although TH expression in extragemmal fibers has been observed in some taste papillae (Dvoryanchikov et al., 2007; Ohman-Gault et al., 2017), we were unable to detect TH expression in extragemmal axons. It is possible that the *Th*-CreER; Rosa^RFP^ mice are not as sensitive as previous immunolabelling experiments. Less than 1/8 the normal dose of tamoxifen (0.03 mg/kg) resulted in recombination and RFP expression in nearly all neurons we observed with typical doses (0.25 mg/kg), suggesting this reporter line is quite responsive. Prior studies have not observed TH+ cells in taste buds themselves (Dvoryanchikov et al., 2007; YA Huang et al., 2008), which is not surprising given how infrequent TH+ taste bud cells were observed in *Th*-CreER reporter mice (Fig. 4). Norepinephrine is produced by taste buds, suggesting it may be involved in neurotransmission within taste buds (Dvoryanchikov et al., 2007; YA Huang et al., 2008), and if TH+ GG neurons produce catecholamines, they may be released into the NTS as well. Expression of TH, however, does not always coincide with catecholamine synthesis. DRG neurons that express TH, for example, do not produce catecholamines because they lack the subsequent synthetic enzymes (Brumovsky et al., 2006). Likewise, TH expression is not a prerequisite for catecholamine production because L-Dopa can be taken up by the L-amino acid transporter and then converted into catecholamines, as is the case for taste bud cells (Dvoryanchikov et al., 2007; YA Huang et al., 2008). Germline deletion of *Th* results in embryonic lethality between E11.5-E14.5 because of the critical role of catecholamines in heart development (Kobayashi et al., 1995; Zhou et al., 1995; Rios et al., 1999). Therefore, analysis of catecholamines in the function of the peripheral taste system will require conditional deletion of *Th* after embryogenesis.

This leads naturally to the question of what physiologic role these TH+ geniculate neurons have in feeding behavior. Current molecular genetic approaches, such as the use of DREADDs to silence these neurons, would also silence TH-expressing taste receptor cells and NTS neurons. Another complication for behavioral studies is the presence of catecholaminergic neurons involved in motivation and reward pathways. Therefore, targeting of TH+ neurons specifically in the GG, and not in other parts of the nervous system, is needed. This could be accomplished with intersectional genetics, as in Figure 5, but instead of diving GFP expression, combined *Th* and *Phox2b* regulates the expression of electrophysiological activators or inhibitors. Approaches such as this will be critical for revealing how specific subpopulations of chemosensory neurons, and their circuits, contribute to the complex behavior of feeding.

## Synthesis

Reviewing Editor: Miriam Goodman, Stanford University Decisions are customarily a result of the Reviewing Editor and the peer reviewers coming together and discussing their recommendations until a consensus is reached. When revisions are invited, a fact-based synthesis statement explaining their decision and outlining what is needed to prepare a revision will be listed below. The following reviewer(s) agreed to reveal their identity: Ann-Marie Torregrossa.

The purpose of this study is to report upon the peripheral connections of a subset of geniculate ganglion neurons that express tyrosine hydroxylase (TH). Reviewers agreed that the anatomical work is carefully generated and identified major and minor concerns that require your attention. Some concerns require additional experiments, while others can be address through improved figure design and reporting (see list below).

Additionally, the manuscript uses a large number of abbreviations that may be off-putting to readers who are not already intimately familiar with the neuroanatomy of the oral cavity or the nucleus tractus solitarius. Please reserve the use of abbreviations to terms that are used throughout the manuscript, write out the full names of structures that are referenced sparingly (e.g. FISH), and revise the manuscript to ensure that all abbreviations are defined upon their first use. Please report the number of animals analyzed in support of each anatomical finding and indicate whether or not the animals included males and females. If a single sex was used, please comment on the limitations of generalizing the findings to other sex.

1. Please provide anatomical markers for images of tissue sections so that readers can more easily discern the plane of section. Rev1 raises a concern regarding the plane of section for images shown in Figure 5 and how the boundaries of the nucleus tractus solitarius (NTS) were determined. Both reviewers recommend that co-labeling with P2X2 would enable you to securely separate tyrosine hydroxylase (TH)+ fibers arising from cranial nevers VII, IX, and X from those intrasolitary TH+ fibers.

2. Relying upon TRPM5 expression as a marker of taste quality was noted as being an overstatement, since the qualities encoded by the target neurons has not been experimentally determined and their remains uncertainty in the field as to how taste quality is encoded.

3. Figure design - As suggested by Rev1, it would be helpful if a given label were to be shown with the same figure throughout the manuscript (even if the fluorophore differs, which could be communicated in the figure legend). Please consider the suggestion to use a palette other than red, green, and blue which can be hard to render (blue and red are particularly hard to see on a black background) and difficult for colorblind individuals to see at all. In this regard, you may find these articles helpful: https://www.nature.com/articles/d41586-021-02696-z and https://thenode.biologists.com/data-visualization-with-flying-colors/research/

4. Statistics - Please report the F statistics and p-values arising from ANOVAs along with the p-values for post-hoc testing. A conventional format for reporting ANOVAs is F(1,24) = 44.4, p = 0.002. Where (1, 24) indicates the degrees of freedom in the test. As noted by reviewers, the p-value provides information regarding the probability that the underlying data were drawn from a single distribution. Lower p-values indicate that this probability is lower not that the size of the effect was larger. To address the latter, you may wish to consider adopting estimation statistics to your analysis - as recommended by eNeuro (https://www.ncbi.nlm.nih.gov/pmc/articles/PMC8021395/)

Reviewer 1

The authors showed the expression of tyrosine hydroxylase (TH) in lingual epithelium and brainstem. The manuscript is clearly written and scholarly. An essential point in the paper is that TH did not overlap with Ret (a marker for mechanosensory neurons in geniculate ganglion neurons), suggesting that TH expression is associated with chemosensory signaling. Labeling for P2X2 would have been a useful way to confirm that TH fibers were, indeed, chemosensory. It would have been useful if the authors had sectioned taste buds in the nasoincisor duct. The chorda tympani (CT) nerve innervates taste buds in fungiform papillae on the anterior tongue, whereas the greater superficial petrosal (GSP) nerve innervates taste buds in the anterior and posterior palate. Taste buds on the anterior palate are located within the nasoincisor duct, whereas taste buds on the posterior palate are located within the soft palate. Taste buds in foliate and circumvallate papillae are innervated, in large part, by the glossopharyngeal nerve, although a small number of neurons CT neurons innervate taste buds in the anterior foliate. Cell bodies of the CT and GSP reside in the geniculate ganglion, whereas cell bodies glossopharyngeal nerve reside in the petrosal ganglion. All three of these cranial nerves terminate in the Nucleus Tractus Solitarius (NTS). The NTS is topographically organized in a manner that roughly resembles the oral cavity. More specifically, nerve fibers with fungiform and nasoincisor duct receptive fields terminate in the most rostral portion of the NTS, whereas nerve fibers with foliate, circumvallate, and soft palate terminate in the more caudal portion of the rostral NTS. Figure 5 shows TH expression in the NTS, but it is difficult to ascertain what is being shown here without high contrast bright field or dark field images or P2X2 labeling of gustatory and visceral afferents from cranial nerves VII, IX, and X, respectively. As shown, it appears that the entire rostral and portion of the caudal NTS is depicted in the photo, and the scale and shape of the TH expression are consistent with this. Gustatory responses in the NTS are primarily recorded from in the first two rostral segments of the NTS (R2 and R3), which is no greater than 600um from the rostral pole. At least 700um of NTS is shown in Figure 5, but the schematic suggests it is only an image of R3. TH neurons are located within the caudal and intermediate NTS, and from Figure 5, it appears that TH+ cell bodies can be identified there. Additionally, it has been shown that catecholaminergic neurons in the caudal visceral NTS project to the rostral NTS. So, the high level of expression of TH+ nerve fibers shown in the NTS here, might arise from cell bodies in the caudal NTS. P2X2 labeling will allow the authors to separate TH positive fibers arising from cranial nerves VII, IX, and X from intrasolitary TH+ fibers.

-Photos in Figures 1 and 2 are remarkable. Because the authors show the expression pattern of TH in taste buds innervated by geniculate and petrosal ganglion neurons, as well as the TH expression in the brainstem, it would be interesting to know what the expression pattern of TH is in the petrosal ganglion. Because the results for ganglion cell bodies are so clear, perhaps an n of 3 would be sufficient.

-Figure 3. “Chorda” was misspelled in panel B.

-Figure 5. The overall shape of the TH expression, plane of section, and the distance from the rostral pole of the NTS to the most posterior portion of the figure makes it appear that the section contains the entire rostral NTS and perhaps a portion of the caudal NTS. In mice, the entire rostral NTS, beginning where the NTS moves away from the fourth ventricle, is approximately 800um. Based on the scale shown here, it appears that the section includes approximately 700-800um of NTS as it moves away from the 4th ventricle. This is, however, only an approximation based on the overall shape of the fluorescent TH expression and scale. High contrast brightfield or darkfield of the same section would reveal the borders of the NTS and boundaries of adjacent nuclei. P2X2 labeling will allow the authors to determine which TH+ positive fibers are gustatory and visceral from those that are intrasolitary TH+ neurons arising from the caudal NTS. Without those additional markers, it is hard to determine what is being shown. Related to this are the dotted lines showing the boundaries of the NTS. What are these based off? Most researchers use darkfield (more recent), Weil stain, or high contrast brightfield to determine the medial border, because the NTS is less myelinated then the laterally adjacent trigeminal nuclei. Based on what I can see here, those certainly aren’t the borders of the NTS...it appears that the dotted lines cross over the midline. Coronal sections would be more useful, as one would be able to see IIV, IX, and X cranial nerves entering from the lateral side of the section and then terminating in the rostral central (gustatory) subdivision of the NTS. If the authors wish to show the NTS in the horizonal plane, then P2X2 labeling and darkfield (or brightfield contrast) will be necessary to show the boundaries of the NTS.

-Page 15: The sentence: “While geniculate ganglion innervation of fungiform and anterior foliate taste buds is predominantly derived from the chorda tympani (CT) nerve, the greater superficial petrosal (GSP) nerve, which also emanates from the geniculate ganglion, innervates taste buds located on the soft palate in the posterior oral cavity (Refs).” The references were not entered. It should also be noted that the GSP innervates taste buds in the nasoincisor duct. The authors should mention somewhere that the lack of information regarding the innervation of TH+ fibers in nasoincisor papillae is a limitation.

-Figure 6. Panel B is clear for all 3 colors overlaid for the fungiform, but not for the soft palate. I would suggest using the same amount of digital zoom in each photo, so that the importance of the effects can be clearly shown. For example, the left bud in panel A for the soft palate has the arrow pointing to a TH positive fiber that close to a Car4 positive taste cell. So, it would be best to show only that bud at a higher zoom to clearly show the effect. The bud on the right could be cropped out of the image. The same goes for panel B...the fungiform bud is shown at a higher zoom than the soft palate buds. It would be better only a single bud was shown and at the same zoom (I assume this is digital zoom, since all images were taken with the same objective).

-The number of mice is often listed for experiments in the body of the manuscript and figure legends. While this is important, it would be useful to know the total number (or average number across animals) of cells/buds that were counted as well.

-Page 11 & figures with graphs: lower p values do not indicate greater significance. Effect sizes analyses would indicate the magnitude of the effects.

-Page 16: The TH+ TBCs - “did not express CAR4” and “were colabeled with TRPM5” does that mean 0% and 100% respectively? It’s never explicitly stated.

-Figures in general: It would be useful if the authors used the same colors for each label for consistency. It would be best if cyan, green, orange, and magenta were used instead of blue, green, and red.

Reviewer 2

This manuscript describes a subset of geniculate ganglion neurons that express Th. Overall the descriptive/anatomical work appears to be very carefully done however the interpretation of the work feels overstated at times. I have out lined some major and minor concerns below.

Major

1- When discussing fig 1 in the results section there is discussion of high and low levels of Th expression. These were quantified as “high” or “low” as making up different percentages of Phox2b neurons. Was Th RNA in these cells quantified by florescence? Where is this quantification? What were the parameters for separating these populations? Please clarify.

2- The presence of TRPM5 and Th in the same cell says nothing about the possible ligands for the cell. The suggestion that they “likely detect bitter, sweet or umami” is an overstatement and was not addressed experimentally.

3- I do not see any evidence that “central projections of TH-expressing oral sensory neurons innervate the rostral nucleus of the solitary tract.” As the authors rightly point out in the paper the NTS is full of TH expressing neurons. It is therefore “impossible to determine where Th+ fibers from gustatory tracts terminate in the NTS”. The evidence presented is that the part of the NTS where one would expect gustatory neurons to terminate has RFP+ cells in it. This is not sufficient to make the claim that the specific neurons of interest are synapsing here. I am not sure what these findings add to the paper as the presence of Th+ neurons in the NTS is unsurprising.

4- Figure 5 would benefit from a lower magnification image inset that allows the reader to see the important anatomical regions on the tissue so it can be determined what level of the NTS the images are from.

Minor-

Table 1 is 4 pages away from its first reference

Abbreviations not defined prior to first use:

CTs (in RNA extraction methods)

FISH (in Quant of geniculate gang methods)

Fig 1A Th, a red arrow is not appropriate when pointing out red stain.

I would not normally comment on writing style but the authors use the phrase “coursing through” to describe what I assume is the presence of a neuron in a nerve? Or the presence of an axon in a brain region? This is an odd phrasing that does not necessarily convey the biology.

Eg. “CT and GSP nerves had RFP+ fibers from the Th+ neurons coursing through them"

"fibers were observed coursing into the rostral NTS"

"RFP+ labeled fibers course through taste buds"

"Phox2B+ neuorns course into taste buds"

The authors suggest they use the reporter mouse because the FISH protocol does not measure protein (in results “our prior experience with FISH...”) but the tag for the reporter is on the TH promotor and also does not measure protein. I think using both is a great idea but neither measure protein so I would edit this section to remove the suggestion that one corrects this deficiency in the other.
