# Supplementary figures and images for: Oral Sensory Neurons of the Geniculate Ganglion That Express Tyrosine Hydroxylase Comprise a Subpopulation That Contacts Type II and Type III Taste Bud Cells
Source: eNeuro. 2022 Oct 12;9(5):ENEURO.0523-21.2022. doi: 10.1523/ENEURO.0523-21.2022 (PMC9581578; doi:10.1523/ENEURO.0523-21.2022)

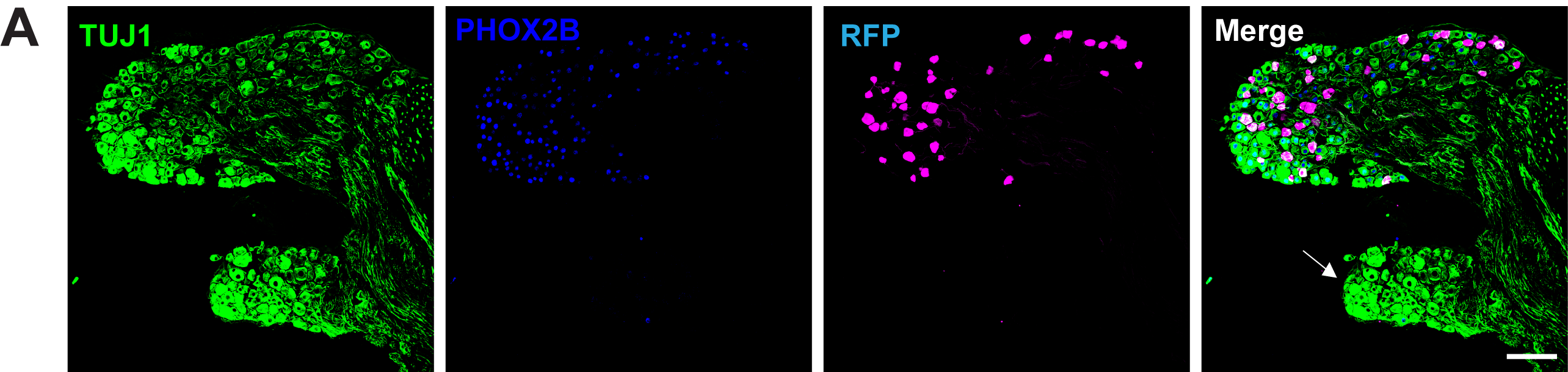

Supplement: Extended Data Figure 1-1 — Th expression in the nodose/petrosal/jugular ganglion complex. Th-CreER; RosaRFP mice were administered tamoxifen and allowed to recover for three weeks. The nodose/petrosal/jugular complex was dissected and sectioned. The sections were immunolabeled with antibodies to TUJ1 (green), PHOX2B (blue), and RFP (cyan). The nodose and petrosal ganglia are often fused together in mice, and the leftmost lobe is the nodose and the rightmost is the petrosal. The jugular ganglion is just anterior along the vagus and is positioned in the lower half of the image, indicated by the white arrow. Some of the PHOX2B+ neurons in nodose/petrosal ganglia are also Th+, consistent with innervation of CV tastebuds by Th-expressing neurons. Similar results were observed in three mice of each genotype. Scale bar: 100 μm. Download Figure 1-1, TIF file. [file enu-eN-NWR-0523-21-s01.tif]
